# Recommended β-lactam regimens are inadequate in septic patients treated with continuous renal replacement therapy

**DOI:** 10.1186/cc10257

**Published:** 2011-06-06

**Authors:** Lucie Seyler, Frédéric Cotton, Fabio Silvio Taccone, Daniel De Backer, Pascale Macours, Jean-Louis Vincent, Frédérique Jacobs

**Affiliations:** 1 Department of Infectious Diseases, Erasme Hospital, Université Libre de Bruxelles, route de Lennik 808, 1070 Bruxelles, Belgium; 2 Department of Clinical Chemistry, Erasme Hospital, Université Libre de Bruxelles, route de Lennik 808, 1070 Bruxelles, Belgium; 3 Department of Intensive Care, Erasme Hospital, Université Libre de Bruxelles, route de Lennik 808, 1070 Bruxelles, Belgium

## Abstract

**Introduction:**

Sepsis is responsible for important alterations in the pharmacokinetics of antibiotics. Continuous renal replacement therapy (CRRT), which is commonly used in septic patients, may further contribute to pharmacokinetic changes. Current recommendations for antibiotic doses during CRRT combine data obtained from heterogeneous patient populations in which different CRRT devices and techniques have been used. We studied whether these recommendations met optimal pharmacokinetic criteria for broad-spectrum antibiotic levels in septic shock patients undergoing CRRT.

**Methods:**

This open, prospective study enrolled consecutive patients treated with CRRT and receiving either meropenem (MEM), piperacillin-tazobactam (TZP), cefepime (FEP) or ceftazidime (CAZ). Serum concentrations of these antibiotics were determined by high-performance liquid chromatography from samples taken before (*t *= 0) and 1, 2, 5, and 6 or 12 hours (depending on the β-lactam regimen) after the administration of each antibiotic. Series of measurements were separated into those taken during the early phase (< 48 hours from the first dose) of therapy and those taken later (> 48 hours).

**Results:**

A total of 69 series of serum samples were obtained in 53 patients (MEM, *n *= 17; TZP, *n *= 16; FEP, *n *= 8; CAZ, *n *= 12). Serum concentrations remained above four times the minimal inhibitory concentration for *Pseudomonas *spp. for the recommended time in 81% of patients treated with MEM, in 71% with TZP, in 53% with CAZ and in 0% with FEP. Accumulation after 48 hours of treatment was significant only for MEM.

**Conclusions:**

In septic patients receiving CRRT, recommended doses of β-lactams for *Pseudomonas aeruginosa *are adequate for MEM but not for TZP, FEP and CAZ; for these latter drugs, higher doses and/or extended infusions should be used to optimise serum concentrations.

## Introduction

Severe sepsis and septic shock are major causes of morbidity and mortality in ICUs [1-3]. Antibiotic treatment, if adequate and given early [4,5], remains of paramount importance to optimise chances of survival [6]. Several studies have shown the crucial impact of the first 24 hours of antimicrobial treatment on outcome [7]. In addition to timing, the chosen antibiotic should target the potential pathogens involved, taking local susceptibility patterns into account. To be effective, the doses given should reach therapeutic concentrations in the blood and at the site of infection [8-10]. Sepsis can significantly alter the pharmacokinetics of antimicrobials and result in subtherapeutic drug concentrations [11,12], potentially contributing to decreased bacterial killing, therapeutic failure and emergence of resistance.

Acute renal failure is a common complication of sepsis. In septic patients, continuous renal replacement therapy (CRRT) is often preferred to conventional haemodialysis because it is better tolerated haemodynamically. However, CRRT can further alter the pharmacokinetics of antibiotics [13]. These changes depend on several variables, such as the ultrafiltrate and dialysate rates, dialysate concentrations and the type of membrane used - each of these variables introducing additional variability in expected drug concentrations [14]. A recent systematic review addressed the importance of all these factors for antibiotic prescription [15], but the most recent recommendations on antibiotic dosing during CRRT [16] were established using evidence from studies that included a limited number of patients, with varying inclusion/exclusion criteria and receiving different types of CRRT [17-20]. Serum measurements were usually performed at steady state, which also limits the extrapolation of results to the early phase of sepsis, during which patients are often haemodynamically unstable. Finally, these recommendations have never been validated in a septic ICU population suffering from multiple organ failure.

The objective of our study was to evaluate whether the recommended doses of broad-spectrum β-lactams [16] result in appropriate serum concentrations in ICU patients with severe sepsis and septic shock receiving CRRT.

## Materials and methods

### Study design, patients and inclusion criteria

This observational, prospective study was conducted between January 2008 and May 2009, in a 35-bed medico-surgical ICU at Erasme Hospital, Brussels (Belgium). The study was approved by the local ethics committee (Comité d'Ethique Hospitalo-Facultaire Erasme-ULB, reference number OM021) and informed consent was obtained from patients or their closest relative. The study was conducted in compliance with the Helsinki Declaration for human research.

Inclusion criteria were as follows: age > 18 years; diagnosis of severe sepsis or septic shock according to standard criteria [1]; acute renal failure treated with CRRT; and receiving at least one of meropenem (MEM), piperacillin-tazobactam (TZP), cefepime (FEP) or ceftazidime (CAZ). All patients fulfilling these criteria were included consecutively. Exclusion criteria were pregnancy, burns and cystic fibrosis. Patients receiving different study drugs successively were included more than once.

### Antibiotic treatment and serum samples

The choice of antibiotic was at the discretion of the clinicians and was based on local guidelines. All included patients received a first dose (loading dose) of 1 g MEM, 4.0/0.5 g TZP, or 2 g FEP or CAZ. The highest daily dose was taken from published recommendations [16] for patients on CRRT, whether on continuous venovenous haemofiltration (CVVH) or continuous venovenous haemodiafiltration (CVVHDF): 1 g twice daily for MEM and 2 g twice daily for FEP and CAZ, whereas for TZP the daily dose was adapted to the European format (that is, 4.0/0.5 g four times daily). Each antibiotic dose was administered as a 30-minute infusion.

Blood samples for drug assays (3 to 4 ml blood) were drawn from the arterial line on the day of inclusion, and then every second day during CRRT treatment when possible. On each sampling day, a series of blood samples was drawn to obtain a pharmacokinetics curve for each dose: immediately before the antibiotic administration (0 hours), and then 1, 2, 5, and 6 or 12 hours (depending on the antibiotic regimen) after the start of the infusion. The exact sampling times were recorded. Blood was collected in plain tubes and centrifuged at 3,000 rpm at 4°C for 10 minutes; the supernatant was separated immediately and kept at -80°C until analyses were performed. Sample series were grouped according to the day of sampling relative to the start of the antibiotic treatment; that is, into the early phase (< 48 hours from the first dose) or the late phase (> 48 hours).

### Continuous renal replacement therapy

CRRT was performed through a double-lumen catheter inserted into a large vein. CVVH or CVVHDF was performed using a Prisma or PrismaFlex machine (Hospal, Meyzieu, France), with an AN69 haemofilter (Gambro Lundia AB, Lund, Sweden). Characteristics of the CRRT were recorded for each patient at each blood sampling time.

### Serum antibiotic analyses

Serum concentrations of all antibiotics were measured in the clinical chemistry department by high-performance liquid chromatography connected to UV spectrophotometry. Briefly, 1 ml methanol was added to 500 μl serum in order to precipitate proteins. The supernatant was separated and evaporated, and the residue was solubilised in 50 mmol/l phosphate buffer, pH 3.8. A 30 μl sample was injected into an Agilent 1200 series chromatograph (Agilent, Diegem, Belgium) equipped with a YMC ODS AQ column (YMC GmbH, Dinslaken, Germany). Antibiotics were separated within 60 minutes in an acetonitrile-phosphate buffer gradient. UV absorbance was monitored at 204 nm for tazobactam and MEM, and at 240 nm for piperacillin, FEP and CAZ. Cefoperazone was used as the internal standard. The limit of quantification was 0.2 μg/ml for tazobactam and 2.0 μg/ml for the other antibiotics. Between-day imprecision was less than 6.5%. As the pharmacokinetics of piperacillin and tazobactam are highly correlated [21] and tazobactam serum concentration curves followed those of piperacillin in our study, only the latter were used in the analysis.

The validation of the analytical method was performed daily, according to the published acceptance criteria for specificity, linearity, accuracy, precision (intra-day (repeatability), inter-day (intermediate precision)) and sensitivity (limit of detection) [22]. Under the described chromatographic conditions, MEM, TZP, FEP and CAZ were identified by sharp and well-resolved peaks. The stability of plasma samples is at least 1 month at -80°C.

### Pharmacokinetic analysis

To determine mean concentrations, each series of time points from each patient was linearised using a logarithmic transformation. Each curve was then reconstructed using fixed time points and the mean concentrations were calculated. The peak concentration was the concentration measured 1 hour after the start of the 30-minute antibiotic infusion. The area under the curve (AUC) for a given drug was calculated from the mean AUCs for each series for a given drug, using the raw concentrations. The AUC was calculated using the trapezoidal rule. Also, AUCs were used to estimate differences in drug exposure between measurements made < 48 hours and > 48 hours from the onset of antibiotic therapy.

The volume of distribution (*V*_d_) for a given drug was the mean of all *V*_d _values from the series taken under that drug, using the following formula applied to the linearised series:

where *C*_0 _is the concentration at the start of the infusion, and:

where *C *is the concentration at time *t*, and *k*_e _is the slope. The total clearance (CL) and the elimination half-life (*t*_1/2_) were calculated with the formulae:

No pharmacokinetic/pharmacodynamic profile was measured for loading doses.

### Pharmacodynamic analysis

In the pharmacodynamic analyses, we considered the minimal inhibitory concentrations (MICs) defined by the European Committee on Antimicrobial Susceptibility Testing as the clinical breakpoints for the pathogens most frequently encountered in nosocomial or ICU infections [23]. As *Pseudomonas aeruginosa *is the most frequent, serious pathogen in the ICU and causes infection associated with the highest mortality rates [24], we used the clinical breakpoint of this pathogen as the target MIC. Sensitivity MIC thresholds for this pathogen are: ≤ 2 μg/ml for MEM, ≤ 16 μg/ml for TZP, and ≤ 8 μg/ml for CAZ and FEP.

Some clinical data suggest that maximal killing of bacteria by β-lactams occurs when serum concentrations are maintained above the MIC of the causative pathogens for extended periods [25-28]. Achievement of maximal bactericidal effect requires 40%, 50% and 60 to 70% coverage of the dose interval for carbapenems, penicillins and cephalosporines, respectively [29]. To achieve optimal bactericidal activity, the adequacy of β-lactam therapy in our study was assessed by measuring the time that the concentration was above more than four times the target MIC. This optimal time that the concentration was above more than four times the target MIC for each drug was considered to be ≥ 40% of the time interval between two doses for MEM, ≥ 50% for TZP, or ≥ 70% for FEP and CAZ [29]. We classified each patient as having an adequate or inadequate pharmacokinetic/pharmacodynamic profile according to the percentage of time during which serum drug concentrations remained above four times the clinical breakpoint for *P. aeruginosa*; that is, ≥ 8 μg/ml for MEM, ≥ 64 μg/ml for TZP, and ≥ 32 μg/ml for FEP and CAZ. Finally, using the concentrations obtained in our population, we calculated the probability of achieving targets for the time that the concentration was above more than four times the target MIC for other MICs found in ICU-isolated Gram-negative bacteria.

### Statistical analysis

Descriptive statistics were performed for all study variables. Discrete variables were expressed as counts (percentage), and continuous variables as means ± standard deviation or median (25th to 75th percentiles). Differences between groups (early versus late) were assessed using Student's *t *test. *P *< 0.05 was considered statistically significant.

## Results

### Patients and series of samples

We included 53 patients, whose demographic and clinical characteristics are presented in Table 1. MEM was given in 17 patients, TZP in 16 patients, FEP in eight patients and CAZ in 12 patients. Sixty-nine series of samples were obtained: MEM, *n *= 22; TZP, *n *= 21; FEP, *n *= 11; CAZ, *n *= 15.

**Table 1:** Patient demographic and haemodynamic data

Patients (*n*)	53
Mean age (years)	62 ± 16
Male/female	30/23
Body mass index	26 ± 8
Medical/surgical admission	31/22
Septic shock	12
ICU stay before inclusion (days)	4 (0 to 33)
Mechanical ventilation	37 (70%)
Co-morbidities	
Chronic obstructive pulmonary disease	10 (19%)
Diabetes mellitus	15 (28%)
Heart disease	19 (36%)
Liver cirrhosis	9 (17%)
Solid organ transplantation	8 (15%)
Malignancy	8 (15%)

Data are expressed as number (percentage), median (range) or mean ± standard deviation.

Nineteen patients were treated with CVVHD and 34 with CVVHDF. The mean blood flow rate was 150 ± 24 ml/minute. The mean ultrafiltration rate was 22 ± 12 ml/kg/hour. Twenty-two of the 53 patients underwent fluid removal; in those patients, the removal rate was 158 ± 140 ml/hour. The mean dialysate rate was 23 ± 9 ml/kg/hour.

### Pharmacokinetic data and pharmacodynamic analysis

Pharmacokinetic data are shown in Table 2. There was a marked inter-individual variation in all pharmacokinetic parameters; *V*_d _was increased for all four drugs when compared with healthy volunteers, with consequently a lower peak concentration. There was no significant impact of the technique (CVVHD versus CVVHDF) on the pharmacokinetics of the studied drugs (data not shown).

**Table 2:** Pharmacokinetic parameters of the four antibiotics

Antibiotic (number of series)	*V*_d _(l/kg)	C_max _(μg/ml)	C_min _(μg/ml)	AUC (mg/hour/ml)	CL (ml/minute/kg)	*t*_1/2 _(hours)
MEM 1 g twice daily (*n *= 22)	0.45 (0.20 to 3.03)	26 (15 to 67)	6 (2 to 11)	134 (61 to 291)	1.15 (0.54 to 3.37)	4.39 (2.61 to 30.5)
TZP 4.0/0.5 g four times daily (*n *= 21)	0.44 (0.22 to 1.72)	138 (36 to 262)	60 (4 to 155)	527 (62 to 1378)	1.15 (0.27 to 6.26)	4.16 (1.05 to 15.3)
FEP 2 g twice daily (*n *= 11)	0.55 (0.33 to 0.94)	43 (28 to 83)	11 (3 to 22)	379 (148 to 483)	1.04 (0.43 to 2.97)	6.17 (3.30 to 22.9)
CAZ 2 g twice daily (*n *= 15)	0.37 (0.22 to 0.84)	78 (54 to 118)	24 (5 to 46)	536 (258 to 906)	0.52 (0.13 to 1.61)	7.74 (2.52 to 33.5)

Data shown as median (minimum to maximum). *V*_d_, volume of distribution; C_max_, peak concentration; C_min_, trough concentration; AUC, area under the curve; CL, total clearance; *t*_1/2_, elimination half-life; MEM, meropenem; TZP, piperacillin-tazobactam; FEP, cefepime; CAZ, ceftazidime.

**Figure 1:**
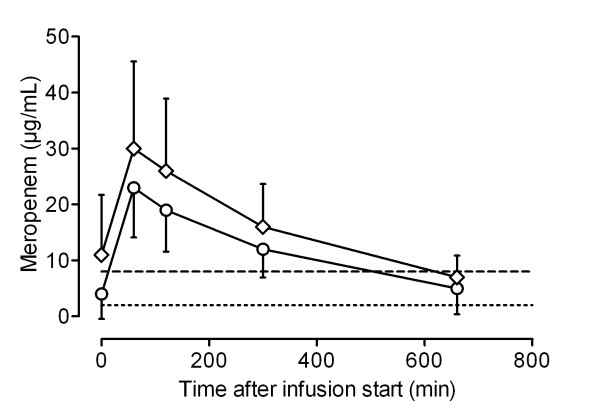
**Pharmacokinetic profile of meropenem for patients receiving continuous renal replacement therapy**. Data shown as mean serum concentrations (with standard deviation) measured in samples taken < 48 hours (circles) and > 48 hours (diamonds) from the start of the treatment. Dotted line, 2 μg/ml; dashed line, 8 μg/ml.

**Figure 2:**
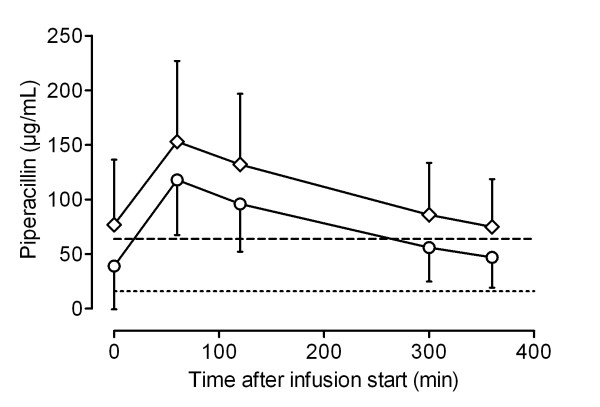
**Pharmacokinetic profile of piperacillin for patients receiving continuous renal replacement therapy**. Data shown as mean serum concentrations (with standard deviation) measured in samples taken < 48 hours (circles) and > 48 hours (diamonds) from the start of the treatment. Dotted line, 16 μg/ml; dashed line, 64 μg/ml.

**Figure 3:**
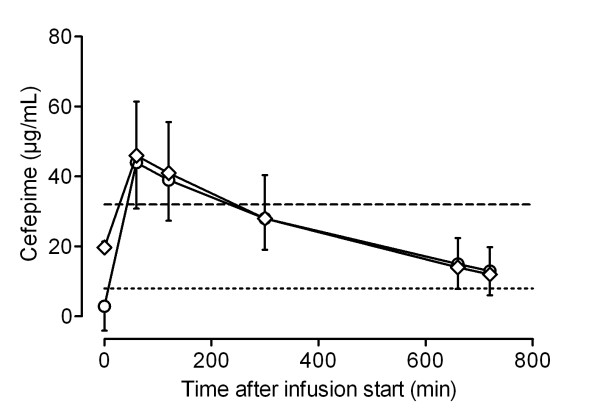
**Pharmacokinetic profile of cefepime for patients receiving continuous renal replacement therapy**. Data shown as mean serum concentrations (with standard deviation) measured in samples taken < 48 hours (circles) and > 48 hours (diamonds) from the start of the treatment. Dotted line, 8 μg/ml; dashed line, 32 μg/ml.

**Figure 4:**
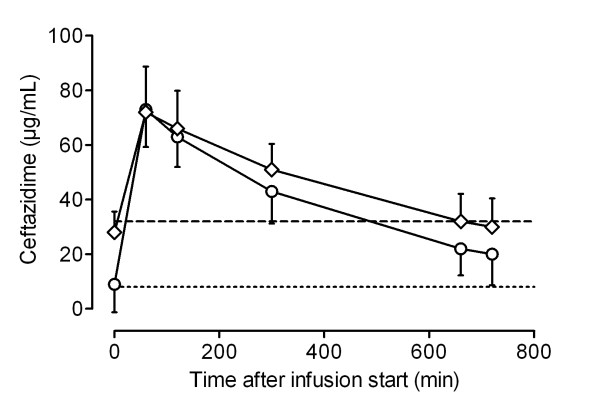
**Pharmacokinetic profile of ceftazidime for patients receiving continuous renal replacement therapy**. Data shown as mean serum concentrations (with standard deviation) measured in samples taken < 48 hours (circles) and > 48 hours (diamonds) from the start of the treatment. Dotted line, 8 μg/ml; dashed line, 32 μg/ml.

Figures 1, 2, 3 and 4 show the concentrations of MEM, TZP, FEP and CAZ over time, separated into early (< 48 hours) and later (> 48 hours) phases of treatment. MEM concentrations were significantly higher in the late phase (> 48 hours) than in the early phase (< 48 hours) (Student's *t *test, *P *= 0.018). Although serum concentrations of TZP, FEP and CAZ were higher after 48 hours of treatment, there was no statistically significant difference between early and later concentrations of these antibiotics.

Pharmacodynamic analyses for each antibiotic against *P. aeruginosa *clinical breakpoints are summarised in Table 3. The pharmacokinetic/pharmacodynamic target was reached in 81% of patients treated with MEM, 71% with TZP and 53% with CAZ but in none of the patients receiving FEP.

**Table 3:** Probability of time the concentration is four times MIC attainment for *Pseudomonas *spp.

Antibiotic, daily dose (number of patients)	Time period (number of series)	PK/PD target attainment (number of series (%))
MEM 1 g twice daily (*n *= 17)	All (*n *= 22)	18 (81%)
	Day < 48 hours (*n *= 7)	5 (71%)
	Days > 48 hours (*n *= 15)	13 (87%)
TZP 4 g four times daily (*n *= 16)	All (*n *= 21)	15 (71%)
	Day < 48 hours (*n *= 12)	8 (66%)
	Days > 48 hours (*n *= 9)	7 (78%)
FEP 2 g twice daily (*n *= 8)	All (*n *= 11)	0 (0%)
	Day < 48 hours (*n *= 7)	0 (0%)
	Days > 48 hours (*n *= 4)	0 (0%)
CAZ 2 g twice daily (*n *= 12)	All (*n *= 15)	8 (53%)
	Day < 48 hours (*n *= 8)	3 (38%)
	Days > 48 hours (*n *= 7)	5 (71%)

Probability of target time that the concentration is four times the minimum inhibitory concentration (MIC) attainment for *Pseudomonas *spp. in the early (< 48 hours) and late (48 hours) phases of sepsis: meropenem (MEM), ≥ 40% of time above 8 μg/ml; piperacillin-tazobactam (TZP), ≥ 50% of time above 64 μg/ml; cefepime (FEP) and ceftazidime (CAZ), ≥ 70% of time above 32 μg/ml. PK/PD, pharmacokinetic/pharmacodynamic.

We calculated the probability of attaining the target time that the drug concentration was above more than four times the target MIC for several MIC values (Table 4). The concentrations would reach this target in > 90% of cases with pathogens for MEM with MICs of one or less, for TZP with MICs of eight or less, and for FEP and CAZ with MICs of two or less.

**Table 4:** Probability of target time the concentration is four times the MIC attainment for various MICs

MIC (μg/ml)	Target concentrations (μg/ml)	MEM (*n *= 22)	FEP (*n *= 11)	CAZ (*n *= 15)	TZP (*n *= 21)
**32**	**128**	0	0	0	3 (14)
**16**	**64**	0	0	0	15 (71)
**8**	**32**	0	0	8 (53)	**19 (90)**
**4**	**16**	9 (41)	7 (63)	13 (87)	21 (100)
**2**	**8**	18 (81)	**10 (90)**	**15 (100)**	21 (100)
**1**	**4**	**22 (100)**	11 (100)	15 (100)	21 (100)
**0.5**	**2**	22 (100)	11 (100)	15 (100)	21 (100)

Data expressed as numbers of series (percentages). Data i**n bold **show targets attained in at least 90% of patients for the indicated minimum inhibitory concentrations (MICs) corresponding to European Committee on Antimicrobial Susceptibility Testing clinical breakpoints for *Pseudomonas aeruginosa*. MEM, meropenem; FEP, cefepime; CAZ, ceftazidime; TZP, piperacillin-tazobactam.

## Discussion

In this population of patients with severe sepsis and septic shock treated with CRRT, we showed that the recommended doses for broad-spectrum β-lactams are generally insufficient to maintain therapeutic serum concentrations greater than four times the MIC of *P. aeruginosa*. In the first 48 hours of treatment, 29%, 34%, 100% and 62% of our patients treated with MEM, TZP, FEP and CAZ, respectively, never reached the pharmacokinetic target. After 48 hours of treatment, the drug concentrations obtained were higher (significantly different only for MEM), but they remained insufficient in many patients. Despite the prolonged *t*_1/2_, we did not find significant drug accumulation for TZP, FEP and CAZ over time. This finding could be due to several concomitant factors that may affect drug concentrations, such as changes in CRRT settings, modification in filter efficacy, renal recovery with additional drug clearance, fluids and vasoactive agent administration with related changes in drug *V*_d _[15]. Also, the smaller number of patients evaluated for these three drugs could have limited this analysis, and therefore larger studies are warranted to address this question. If we apply our results to other MICs, the observed concentrations for all antibiotics were adequate in 90% of patients only for MICs lower than the clinical breakpoint of *Pseudomonas *spp., which correspond to MICs of sensitive *Enterobacteriacea*.

Reaching high target concentrations early in the course of treatment seems particularly important in severely ill patients [30], especially given the heterogeneous nature of these patients [31]. In such patients, the pharmacokinetics is altered both in terms of distribution (sepsis itself can modify the *V*_d_, resuscitation measures and nutritional factors) and of elimination (drains, altered metabolism and clearance changes). The higher *V*_d _in the initial phase of sepsis has been previously described in studies on aminoglycosides [32,33] and vancomycin [34]. We recently demonstrated an increased *V*_d _and a high variability of serum antibiotic concentrations in ICU patients with severe sepsis and septic shock [14].

Effective cure of infection in ICU patients can be compromised for other reasons. First, ICU patients are frequently immunosuppressed because of underlying diseases, treatments, or other medical interventions [28]. Impairments in neutrophil and monocyte/macrophage functions are common in critically ill patients and may play a role in the increased risk of developing sepsis and in the severity of the infection. Secondly, bacterial loads can be particularly high; for example, in ventilator-associated pneumonia, intra-abdominal abscesses or peritonitis. Finally, resistant bacteria can be selected by prior antimicrobial treatment or through nosocomial transmission. For the above reasons, high concentration targets may be preferable in difficult-to-treat infections such as those caused by *Pseudomonas *spp., which are associated with the highest mortality rates.

β-lactam antibiotics have long been known to have time-dependent antibacterial activity [35]. The time above the MIC of the infecting organism is the best parameter to reflect the efficacy of β-lactams [36]. *In vitro *killing curve studies have shown that the β-lactam killing activity was rapidly saturated at concentrations corresponding to four times the MIC, so that greatly increasing antibiotic concentrations (that is, above eight or 16 times the MIC) did not kill bacteria more rapidly or more extensively [29,37]. In animal models, maximal bacterial killing was obtained with drug concentrations of four or five times the MIC [38]. Unfortunately, there are no data comparing the efficacy of different therapeutic endpoints in the clinical setting. Microbiological success, but not clinical cure, was significantly correlated with the proportion of the dosing interval when FEP concentrations exceeded four times the MIC in human infections [39]. Based on these limited data, we selected specific pharmacodynamic endpoints to assess the adequacy of β-lactam concentrations in our study. The time during which the serum concentration should remain above a threshold concentration (40 to 100%) and the optimal threshold concentrations (one to four times the MIC), however, are still controversial [40]. In the present study, we deliberately chose to consider the clinical breakpoint of *P. aeruginosa*, commonly isolated in ICU patients with higher MICs than most other *Enterobacteriaceae *and associated with the highest mortality rates [41].

### Continuous renal replacement therapy and antibiotic concentrations

Studies on serum concentrations of broad-spectrum β-lactams in patients undergoing haemofiltration/haemodiafiltration have shown varying results, but included small and variable patient populations, CRRT types, and MIC target values. Studies of patients on CRRT receiving MEM have proposed doses ranging from 500 mg every 12 hours [42,43] to 1 g every 12 hours [20,44]. In nine septic patients, the *V*_d _was 29.5 ± 2.7 l (*V*_d _= 39.7 l (14.8 to 184.7 l) in the present study), the AUC was 118.0 ± 15.8 mg/hour/l, the total CL was 143.7 ± 18.6 ml/minute (total CL = 88.7 ml/minute (43.2 to 205.7 ml/minute) in the present study) and the *t*_1/2 _was 2.33 ± 0.38 hours [42]. On the basis of a literature review, Trotman and colleagues suggested doses of 1 g every 12 hours for CVVH or CVVHDF [16]. In our study, we found using these recommended doses that adequate concentrations of MEM were obtained only for pathogens with an MIC up to 1 μg/ml.

For TZP, doses of 4.0/0.5 g every 12 hours were reported to be insufficient for *Enterobacteriaceae *and *Pseudomonas *spp. in six patients on CVVH or CVVHDF [45]; these authors suggested adding an extra dose daily. The adequacy of 4.0/0.5 g three times daily was reported in a study of nine patients on CRRT who maintained concentrations > 125 μg/ml for the whole time interval [46]. Simulations to reach a MIC of 32 μg/ml have also been obtained with 4.0/0.5 g every 12 hours or 2.0/0.25 g every 8 hours [19]. In this latter study, a mean *V*_d _of 0.31 ± 0.07 l/kg, a median piperacillin total CL of 47 ml/minute (26 to 220 ml/minute) and a mean *t*_1/2 _of 4.3 ± 1.2 hours were found [19]. These results were comparable with those found in our study. Even with a slightly higher dose of TZP (4.0/0.5 g every 6 hours in our study) than that proposed by Trotman (3.0/0.375 g every 6 hours), however, our patients did not reach adequate concentrations for higher MICs.

Malone and colleagues confirmed that FEP was eliminated well by CRRT but that 2 g daily would be sufficient for MICs < 8 μg/ml [17]; higher MICs may need 4 g/day. In this study, the authors reported a *V*_d _of 0.46 ± 0.14 l/kg, a CL of 0.40 ± 0.09 ml/minute/kg, and a *t*_1/2 _of 12.9 ± 2.6 hours [17], similar to our study. Similarly, results in one study of six patients on CVVHDF receiving 2 g twice daily were judged satisfactory given the trough concentrations of 17.7 μg/ml [47]. Despite using these doses in our study, FEP showed the worst results, except for very susceptible organisms. Studies on CAZ have also given discordant results: Matzke and colleagues proposed 250-750 mg/day depending on the residual renal function [18]. Other authors opted for maintaining the usual doses of 2 g three times daily for susceptible bacteria, and even suggested 3 g three times daily for higher MICs [48]; these authors reported maximum and trough concentrations comparable with those found in our study, with mean pharmacokinetic parameters as follows: peak concentration = 58.2 ± 11.6 mg/l, AUC = 344 ± 51.6 mg/hour/l, total CL = 98.7 ± 13.2 ml/minute and *t*_1/2 _= 4.3 ± 0.6 hours.

According to all these data, optimisation of β-lactam regimens should be considered in cases of infection by less susceptible pathogens in septic patients during CRRT. Over the past couple of years, studies have emerged on prolonged [49] or continuous infusions of β-lactam antibiotics [11,21,49-51] to increase their antibacterial activity. Nevertheless, data on the efficacy of prolonged or continuous drug infusions during CRRT are scarce [52,53] and further studies are necessary to evaluate this strategy in this setting. Importantly, when drug monitoring is performed in ICU patients, β-lactam regimens were modified in most patients, either because of insufficient concentrations or antibiotic accumulation [54]. Therapeutic drug monitoring should therefore be performed in this setting whenever possible.

### Advantages and limitations

Unlike other studies using well-controlled steady-state conditions, our study was conducted in a real-life setting in acutely ill patients in the ICU. We studied consecutive patients without exclusion. All patients had severe sepsis (with or without septic shock) and were included in the early stage of sepsis. Due to their unstable condition, CRRT parameters were modified many times in the course of each antibiotic treatment. This contrasts with studies performed in selected stable patients with unchanged CRRT parameters, and makes our study a close reflection of everyday practice, rendering our results more relevant and applicable to severely ill patients in mixed ICUs.

The present study has some limitations. First, as only free drug is the active moiety, it has been recommended that all pharmacokinetic/pharmacodynamic indices should be referenced to the unbound (free) fraction of the drug, especially for some drugs such as piperacillin that has 20 to 30% protein binding. Nevertheless, in the case of FEP, differences in total and free concentrations are between 2 and 6%. We considered that the protein binding was negligible for all four antibiotics and used the total concentration of each antibiotic in the analyses; however, we cannot present any data on free drug levels. Second, CRRT use was not standardised and contributed to the large variability of pharmacokinetic parameters observed over the study period. Third, we did not simulate any dose regimens that would have resulted in adequate drug concentrations in our cohort of patients, and a prospective study with dose adaption during CRRT is needed in this setting. Moreover, it is possible that critically ill patients undergoing CRRT would also need a larger than recommended loading dose for β-lactams, as recently shown for a large septic population [14]; however, no loading dose analysis was included in our study. Finally, we did not collect microbiological data or daily severity scores, such as daily Sequential Organ Failure Assessment, and we cannot provide any data on the relationship between drug concentrations and clinical efficacy or evolution of organ dysfunction.

## Conclusions

At the onset of sepsis in patients receiving CRRT, we suggest that similar β-lactam doses to those used in the absence of renal failure should be given during the first 48 hours of therapy: MEM can be given at a dose of 1 g three times daily, TZP at a dose of at least 4 g/0.5 g four times daily, and FEP and CAZ at doses of 2 g three times daily. Dose reduction should be considered thereafter to avoid drug accumulation. Considering the large pharmacokinetics variability, therapeutic drug monitoring of β-lactams should be performed to optimise antibiotic efficacy.

## Key messages

• In the first 48 hours of treatment for patients with sepsis receiving CRRT, antibiotic concentrations were inadequate to maintain therapeutic serum concentrations greater than four times the MIC of *P. aeruginosa *in 29%, 34%, 62% and 100% of patients receiving MEM, TZP, CAZ and FEP, respectively, if given at the current recommended doses for CRRT.

• For patients on CRRT, we recommend using the same antibiotic doses for MEM, TZP, FEP and CAZ as those used in patients without renal failure for the first 48 hours of treatment.

• There may be some accumulation of antibiotic after the first 48 hours of treatment in patients on CRRT, justifying use of drug monitoring whenever possible.

## Abbreviations

AUC: area under the curve; CAZ: ceftazidime; CL: total clearance; CRRT: continuous renal replacement therapy; CVVH: continuous venovenous haemofiltration; CVVHDF: continuous venovenous haemodiafiltration; FEP: cefepime; ICU: intensive care unit; MEM: meropenem; MIC: minimum inhibitory concentration; TZP: piperacillin-tazobactam; *t*_1/2_: elimination half-life; *V*_d_: volume of distribution.

## Competing interests

FST, FJ and J-LV have received honoraria for lectures from Astra Zeneca. J-LV is also on the speakers list of GlaxoSmithKline. The other authors declare that they have no competing interests.

## Authors' contributions

FJ, FST and FC contributed to the conception and design of the study protocol. LS, FST, FJ DDB and J-LV participated in the coordination of the study and data collection. FC and PM performed the pharmacokinetics analyses and contributed to the analysis and interpretation of the data. All authors were involved in drafting the manuscript or revising it critically for important intellectual content. All authors read and gave final approval of the present version of the manuscript to be published.
